# Detection of abortifacient agents in domestic ruminants, with a specific focus on *Coxiella burnetii*

**DOI:** 10.12688/openreseurope.19270.2

**Published:** 2025-06-30

**Authors:** René van den Brom, Susan Neale, Elsa Jourdain, Anneleen Matthijs, Marcella Mori, Elodie Rousset, Katja Mertens-Scholz, Tom N. McNeilly, Ana Hurtado

**Affiliations:** 1 Small Ruminant Health Department, Royal GD, Deventer, 7400 AA, The Netherlands; 2 Penrith Veterinary Investigation Centre, Animal and Plant Health Agency (APHA), Cumbria, CA11 9RR, UK; 3 University of Clermont Auvergne, INRAE, VetAgro Sup, UMR EPIA,, Saint-Genès Champanelle, France; 4 Sciensano, Belgian Institute for Health, Brussels, Belgium; 5 ANSES, Sophia Antipolis Laboratory, Animal Q fever Unit, Sophia Antipolis, France; 6 Friedrich-Loeffler-Institut, Institute of Bacterial Infections and Zoonoses, Jena, 07743, Germany; 7 Moredun Research Institute, Penicuik, EH26 0PZ, UK; 8 Animal Health Department, NEIKER – Basque Institute for Agricultural Research and Development, Bizkaia, 48160 Derio, Spain

**Keywords:** ruminants, abortion, diagnostics, Coxiella burnetii

## Abstract

Q fever is a widespread zoonotic disease caused by the bacterium, *Coxiella burnetii*. In ruminants, *C. burnetii* can cause abortions, stillbirths, premature births, and weak offspring. As part of the EU-funded Q-Net-Assess International Coordination of Research on Infectious Animal Diseases (ICRAD)-project, aimed at generating the most comprehensive understanding of *C. burnetii* genetic variation to date and determining the implications of this genetic variation for zoonotic risk, pathogenicity and control of *C. burnetii* infection, we have reviewed the protocols reported by the different project partners and/or countries to diagnose abortion in domestic ruminants. As a result of this review, we have developed guidelines for the detection of abortifacient agents in domestic ruminants, with a special focus on *C. burnetii*. They include a description of the essential and complementary samples needed for a definitive diagnosis, the analytical techniques to be used, and the interpretation and validity of each type of sample and technique. The most comprehensive diagnostic approach to identify an infectious agent as the cause of abortion in ruminants would include histopathology, including immunohistochemistry (IHC), on the fetus and placental membranes, complemented by bacteriology, serology, and real-time PCR analyses of different types of samples. For the specific diagnosis of *C. burnetii* as the causative agent of abortion, we provide guidelines based on expert opinions for the interpretation of laboratory test results in relation to their diagnostic value.

## Plain Language Summary

Q fever, caused by the bacterium *Coxiella burnetii*, can be transmitted from animals to humans. It may cause reproductive issues in animals, such as cows, sheep, and goats, including miscarriages and weak offspring. The EU-funded Q-Net-Assess project aims to understand the genetic variations in *C. burnetii* and their impact on disease spread, severity, and control. Researchers in this project reviewed European methods for diagnosing miscarriages in these animals and created guidelines to identify causative agents, focusing on *C. burnetii*. The guidelines specify the necessary samples, diagnostic techniques, and interpretation of the results. The best diagnostic approach involves examining fetal and placental tissues using immunohistochemistry (IHC) supported by bacteriology, serology, and real-time PCR tests. For diagnosing *C. burnetii*, expert advice is provided to interpret the lab results.

## Introduction

Abortion and stillbirths in ruminants are a major cause of economic losses for livestock producers. The causes can be multifactorial, including infectious and non-infectious. Non-infectious factors that can influence fetus viability are hormonal fluctuations, genetic abnormalities, congenital malformations, nutritional deficiencies, and trauma, but also pharmacological, environmental and toxic exposure may cause abortion ( Givens & Marley, 2008; Hecker *et al.*, 2023; Mee, 2023). Infectious causes include a wide range of viral (e.g. pestiviruses, Rift Valley fever), protozoal (e.g. *Neospora caninum*, *Toxoplasma gondii*), bacterial ( *Brucella melitensis*, *Chlamydia abortus*, *Coxiella burnetii*, *Listeria* spp., *Salmonella* spp.), and fungal pathogens. Of these, some are regarded as abortifacient agents, e.g. *B. melitensis*, *N. caninum,* while opportunistic bacteria such as *Salmonella* spp. and *Listeria* spp. may more incidentally cause abortion ( Hecker *et al.*, 2023). The distribution and incidence of infectious causes of abortion may vary in different parts of the world ( Hecker *et al.*, 2023; Mee, 2023; Thomas *et al.*, 2022). Identifying the etiological cause is very useful to prevent future abortions and stilbirths, but it remains a challenge and less than 40–50% of the cases investigated are attributed to a specific etiological agent.

Some important abortifacient agents have zoonotic potential; this is the case for *Coxiella burnetii*, the causative agent of Q fever, a widespread zoonotic disease. Infections caused by *C. burnetii* are referred to as coxiellosis in animals and Q(uery) fever in humans ( Plummer *et al.*, 2018; WOAH, 2018). However, since the publication of case definition guidelines in the WOAH Terrestrial Code in 2024, the preferred terminology promoted by WOAH is 'Infection by Coxiella burnetii' ( WOAH, 2024). Clinical symptoms in humans range from flu-like symptoms to persistent and potentially fatal infections, although asymptomatic infections and flu-like symptoms are more common than the latter ( Angelakis & Raoult, 2010; Eldin *et al.*, 2017; Maurin & Raoult, 1999). Transmission is usually airborne and results from inhalation of infectious dust particles or aerosols ( Angelakis & Raoult, 2010; Clark & Soares Magalhães, 2018). Ruminant livestock, particularly sheep and goats, are the primary sources of human infections, although *C. burnetii* can infect a wide range of other animals, including wildlife, domestic birds, and pets ( Angelakis & Raoult, 2010; Celina & Cerny, 2022; EFSA, 2010; Marrie *et al.*, 1988; Maurin & Raoult, 1999; WOAH, 2018). It can also infect ticks, which may act as vectors and reservoirs, although their role in the epidemiology of the disease has likely been overestimated in some studies owing to diagnostic difficulties in distinguishing *C. burnetii* from *Coxiella*-like endosymbionts within tick populations ( Duron *et al.*, 2015; Jourdain *et al.*, 2015; Körner *et al.*, 2021; Yessinou *et al.*, 2022).

In ruminants, *C. burnetii* can cause abortion, stillbirth, premature birth, and weak offspring (the ASPW complex). Coxiellosis may therefore lead to significant economic losses, particularly in small ruminants (goats and, to a lesser degree, sheep), in which abortion waves affecting all breeding females of a herd may occur ( EFSA, 2010). In cattle, most infections are asymptomatic and abortions are sporadic. Disorders such as subfertility, endometritis/metritis, and retained fetal membranes (RFMs) have been associated with *C. burnetii* infection status in cattle, although direct evidence linking *C. burnetii* infection with these disorders is currently lacking ( Agerholm, 2013). Furthermore, a recent systematic review concluded that although associations between *C. burnetii* infection status and RFMs and infertility/subfertility have been suggested, the association between endometritis/metritis in cattle and *C. burnetii* infection is far from clear ( Gisbert *et al.*, 2024).

Overall, the host range and infection outcome are highly variable. Although host factors may contribute to this outcome, our understanding of how *C. burnetii* genotype contributes to this variation is limited. Currently, the main genotyping methods used for *C. burnetii* generate limited genomic information and are difficult to standardize between laboratories. Whole genome sequencing (WGS) provides comprehensive genetic information and is easily standardized between laboratories and consequently, WGS has revolutionized the molecular epidemiology and surveillance of many zoonotic pathogens. However, due to difficulties in isolating the bacteria from field samples, few *C. burnetii* strains are currently available for WGS.

In 2023, a consortium consisting of partners from Belgium, France, Germany, Spain, the Netherlands, and the United Kingdom began with the EU-funded project Q-Net-Assess as part of the ICRAD (International Coordination of Research on Infectious Animal Diseases) initiative. The project aims to generate the most comprehensive understanding of *C. burnetii* genetic variation and to determine the implications of this genetic variation for zoonotic risk, livestock pathogenicity, and control of *C. burnetii* infection ( https://q-net-assess.com/).

Work package (WP) 1 is aimed at collecting *C. burnetii* positive field samples from a range of hosts and environmental sources to obtain strains for isolation methods optimisation (WP2), WGS (WP3), as well as phenotypic and genotypic analyses (WP4). The collection included both animal and human samples and was built from archived and prospectively collected samples. Archived samples were obtained from the biobanks of human and animal origin samples from different consortium partners. Prospective animal sample collection mainly includes samples from cases of abortion in ruminants, as well as other types of samples from ruminant farms, such as bulk tank milk (BTM), environmental samples, and wildlife whenever available. Prospective sampling of *C. burnetii* associated with abortions in ruminants is being performed using coxiellosis surveillance systems already in place and under local mandatory monitoring programs in some of the participating countries. As part of WP 1, laboratory procedures used at different laboratories for routine testing for concurrent abortifacients were shared and compared to ensure that they all comply with the minimum requirements for the investigation of *C. burnetii* and share equivalent criteria for case definition.

This manuscript compiled the information gathered from a review of the protocols used by different project partners and/or countries. It identifies the essential and complementary samples needed for an accurate diagnosis, describes the interpretation and validity of each type of sample and analytical techniques to detect *C. burnetii* as the causative agent of the abortion, and establishes the minimum requirements for *C. burnetii* investigation and the criteria for case definition.

## Methods

At the commencement of the project, all partners involved were requested to submit the protocols applied in their institute/country to investigate abortions in ruminants. Partners included national reference laboratories for *C. burnetii* in animals (ANSES, APHA, FLI) and both animals and humans (Sciensano), as well as research organizations (APHA, INRAE, MRI, NEIKER, and Royal GD), all specializing in animal health, including coxiellosis. Some of these institutes perform (NEIKER, APHA, MRI) or even centralize the diagnostic process in their country (Royal GD), while others (ANSES, FLI, INRAE, Sciensano) do not routinely investigate abortions in ruminants and have reported the methods collected from networks of veterinary diagnostic laboratories in their respective countries. During the in-person kick-off meeting of the project (31 May 2023), concept protocols for abortion diagnostics were discussed. Different types of samples, diagnostic tools used, and agents investigated were inventoried using standardized templates, which were the drafts of the Table 2– Table 3 that are finally incorporated within this manuscript. Partners gathered this information during discussions with national institutes involved in abortion diagnostics. These protocols were completed by partners and discussed in four online project working group meetings (7 July 2023, 13 October 2023, 12 January 2024, 12 April 2024). After these meetings, a preliminary draft report was written and shared with partners, which was discussed during the annual internal in-person project meeting (30 May 2024). Based on the input from all partners, the final version of the report was written, subsequently discussed with all partners during two virtual working group meetings (12 July 2024 and 11 October 2024) and then approved by all partners before submission (December 2024). The protocols presented here are those in use as of February 2025.

## Results

### 3.1 Investigation of ruminant abortion submissions

The analysis of the ruminant abortion protocols shared by the consortium partners showed that the pathogens investigated as the possible cause of abortion in ruminants, and the techniques used varied among different institutes and/or countries. The agents that were investigated included viruses (Schmallenberg virus, pestiviruses, bovine herpes virus), bacteria ( *C. burnetii*, *Chlamydia abortus*, *Listeria* spp., *Campylobacter* spp., *Salmonella* spp., *Leptospira* spp., *Mycoplasma* spp. and other abortifacient bacteria which can be detected through aerobic and anaerobic culture), parasites ( *Neospora caninum* and *Toxoplasma gondii*), and mycotic agents ( Table 1). Testing for certain agents is restricted to specific ruminant species (e.g., *T. gondii* in sheep and goats; bovine herpesvirus only in cattle) or specific epidemiological contexts (i.e., presence of agents in a certain region) and therefore differs among countries, while other pathogens are the subject of EU and/or country-specific legislation (i.e., *Brucella*). The pathogens investigated and the diagnostic techniques used by each institute/country are presented in Table 2a– f. Protocols usually include a panel of pathogens that are routinely investigated, which vary according to the specific epidemiological context of each region, plus additional agents that are only investigated when specific clinical signs or pathological findings are observed or a diagnosis is not achieved using the standard protocol. Some countries follow standardized protocols that include the animals to be sampled, the type of sample(s), the tests to be performed, and the interpretation of the results for each disease (e.g. the Observatory of Causes of Abortions in Ruminants (OSCAR) protocol in France); others define (mandatory) tests supported by the government (e.g. the Belgian “Abortion Protocol” funded by the Federal Agency for the Safety of the Food Chain - FASFC). In other countries (e.g., Germany and Spain), there are no general guidelines for the diagnosis of abortions in ruminants. A combination of histopathology, bacterial culture, serology, and/or real-time PCR is used by all partners, using specific approaches that vary depending on the targeted etiological agent.

**Table 1.** Pathogens investigated as possible cause of abortion in ruminants in the different institutes and/or countries.

	Reporting partner	ANSES	APHA	FLI	NEIKER	Royal GD	Sciensano
	Scope	France ^1^	UK	Germany ^2^	Spain ^3^	The Netherlands	Belgium ^5^
**Pathogens**	***Coxiella burnetii***	**✓**	**✓**	**✓**	**✓**	**✓**	**✓**
	***Anaplasma* sp.**	**✓**	**(✓)**	**NR**	**(✓)**	**NR**	**(✓)**
	***Brucella* sp.**	**✓**	**✓**	**✓**	**✓**	**NR ^4^**	**✓**
	***Campylobacter* sp.**	**✓**	**✓**	**✓**	**(✓)**	**✓**	**✓**
	***Campylobacter fetus* subsp. ** ***venerealis***	**✓**	**(✓)**	**✓**	**(✓)**	**NR ^4^**	**NR**
	***Chlamydia abortus***	**✓**	**✓**	**✓**	**✓**	**✓**	**✓**
	***Leptospira* sp.**	**✓**	**✓**	**✓**	**✓**	**✓**	**(✓)**
	***Mycoplasma* sp.**	**NR**	**(✓)**	**(✓)**	**(✓)**	**✓**	**NR**
	***Tritrichomonas foetus***	**NR**	**(✓)**	**(✓)**	**(✓)**	**NR**	**NR**
	***Ureaplasma* sp. **	**NR**	**(✓)**	**NR**	**(✓)**	**NR**	**(✓)**
	**Other bacteria (e.g., *Salmonella* sp., ** ***Listeria* sp., *Yersinia* sp., *Trueperella *** ***pyogenes*)**	**✓**	**✓**	**✓**	**✓**	**✓**	**✓**
	***Neospora caninum***	**✓**	**✓**	**✓**	**✓**	**✓**	**✓**
	***Toxoplasma gondii***	**✓**	**✓**	**(✓)**	**✓**	**✓**	**✓**
	**Bluetongue virus**	**(✓)**	**(✓)**	**(✓)**	**NR**	**(✓)**	**(✓)**
	**Bovine herpesvirus**	**NR**	**(✓)**	**✓**	**✓**	**✓**	**(✓)**
	**Pestivirus (Border disease virus-BD; ** **Bovine viral diarrhoea virus-BVD)**	**✓**	**✓**	**✓**	**✓**	**✓**	**✓**
	**Schmallenberg virus**	**(✓)**	**(✓)**	**✓**	**(✓)**	**(✓)**	**(✓)**
	**Mycoses (particularly *Aspergillus* sp.)**	**✓**	**✓**	**(✓)**	**(✓)**	**✓**	**✓**

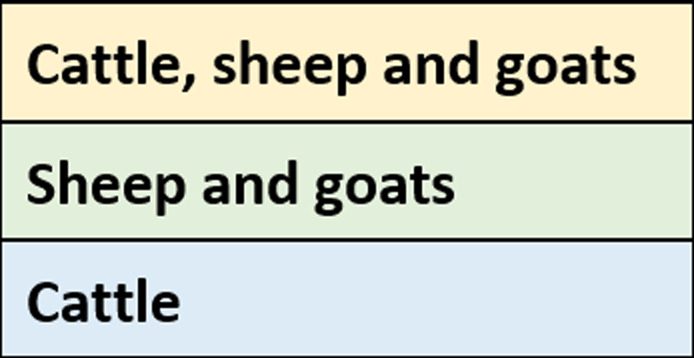
 (√) In brackets, pathogens are not included in all cases in the standard abortion protocol; they are only investigated in particular circumstances based on specific clinical signs, epidemiological context, or post-mortem findings, and optionally, when a diagnosis is not achieved with the standard protocol. For example, *Tritrichomonas foetus* and *Campylobacter fetus* subsp. *venerealis* may be investigated in cases of infertility; Bluetongue virus and Schmallenberg virus if the fetus shows typical abnormalities, and the investigation of *Anaplasma*, *Mycoplasma* and *Ureaplasma* may be based on history and/or clinical findings. NR refers to those not routinely investigated, but tests can be performed if needed. ^1^ The French laboratory network follows the OSCAR (Observatory of Causes of Abortions in Ruminants) protocol, a prototype scheme for differential diagnosis, that is voluntary. Pathogens not included (in brackets or denoted NR) were investigated in particular circumstances, as described above. The OSCAR protocol aims to identify infectious causes of abortion in ruminants (cattle, sheep, and goats) using harmonised diagnostic recommendations at the national scale. The network involves not only laboratories but also the entire chain of involved partners, starting from sampling on farms: the key stakeholders involved included GDS France, the National Animal Health Surveillance Platform (Plateforme ESA), farmers, practicing veterinarians, veterinary diagnostic laboratories, departmental veterinary services, and technical and research institutes. It is important to note that OSCAR's annual report does not provide nationwide surveillance data, as the system is still under development; therefore, the collected data are not representative of the entire French territory. ^2^ There are no general guidelines for the diagnosis of abortions in Germany. Specific regulations exist for certain pathogens, e.g. Brucella. The panel described here is based on submission recommendations from seven state veterinarian diagnostic laboratories and five private diagnostic laboratories. ^3^ There are no general guidelines for the diagnosis of abortion in Spain. There are specific laws and regulations for certain pathogens (e.g., *Brucella*). The panel described here was based on the protocol used at NEIKER (Basque Country, Spain) for the investigation of abortion cases submitted for diagnosis. ^4^ The Netherlands is free of brucellosis, *Campylobacter fetus* subsp. *venerealis* and *Tritrichomonas foetus*; therefore, these pathogens are not included in the ruminant abortion investigation protocol. Brucellosis has been investigated in cattle by compulsory testing of aborted cattle. For small ruminants, sheep and goats from 1,475 farms are tested annually using the Rose Bengal Test. ^5^ In Belgium, the 'Abortion Protocol' is implemented for the diagnosis of ruminant abortions via the laboratories of ‘Dierengezondheidszorg Vlaanderen’ ( DGZ) and ‘Association Régionale de Santé et d’Identification Animales’ ( ARSIA). Beyond mandatory tests defined by disease-specific legislation (e.g., for brucellosis and coxiellosis), the basic protocol encompasses a comprehensive panel of other tests aimed at identifying the most common infectious causes of abortion in ruminants. See Table 2e for specific details. The panel of tests may change depending on the epidemiological situation, and the FASFC bears analysis costs. Next, DGZ/ARSIA offers additional tests for abortion diagnosis subject to a fee. The additional tests offered by the two laboratories may differ slightly.

**Table 2a.** Abortion diagnosis in France (reporting partners: ANSES and INRAE).

Pathogen / Test ^1^	Histopathology	Bacteriology (culture)	Serology	PCR or RT-PCR
***Coxiella burnetii***			S	VS; P; F; FSC
***Anaplasma* sp.**			S	- *A. marginale*: B - *A phagocytophilum*: B; P; VS
***Brucella* sp.**		VS (if serology positive)	S (mandatory)	VS (if serology positive)
***Campylobacter fetus* (subsp. ** ***fetus* and v *enerealis* - cattle)**				ES
***Chlamydia abortus***			S	VS; P; F; FSC
***Leptospira* sp.**			S	P; FSC; VS
**Other bacteria (e.g. ** ***Salmonella* sp., *Listeria* sp., ** ***Yersinia* sp., *Trueperella* sp., ** ***Bacillus* sp.)**		*Salmonella* sp. & *Listeria* sp.: FSC; F (spleen, liver, brain); VS; P	*Salmonella* sp.: S (SAT)	*Salmonella* sp.: FSC (primarily); F (spleen, liver); VS
***Neospora caninum***	F (brain)		S	F (brain)
***Toxoplasma gondii***			S	F (brain, primarily; umbilical cord, possible); P)
Bluetongue virus*				B
**Pestivirus (BD / BVD)**			S	- BD: B (stunted or sick newborns); F (spleen primarily, brain, liver); P - BVD: F (spleen); P
Schmallenberg virus*	Congenital malformations			F (brain primarily, blood, spleen)
**Mycoses (particularly ** ***Aspergillus*)**	If culture positive	P, FSC		

B, maternal blood (in EDTA-tube); ES, endocervical swab; F, fetus; FSC, fetal stomach contents; P, placenta (taken from the uterus); S, maternal serum; VS, vaginal swabs. SAT, serum agglutination test. ^1^ According to the OSCAR protocol except for some agents, indicated here by an asterisk, considered as abortifacients in specific contexts.

**Table 2b.** Abortion diagnosis in Great Britain, UK (reporting partner: APHA).

Pathogen / Test	Histopathology	Bacteriology		Serology (ancillary test)	PCR or RT-PCR
		Culture	MZN		
***Coxiella burnetii***	P		P; FSC	B (ELISA)	P; FF
*Anaplasma* sp. *	F				B
***Brucella* sp.**		FSC; P	P; FSC	B (RBT, ELISA)	
***Campylobacter* sp.**		FSC			
***Campylobacter fetus* subsp.** venerealis * (cattle)		FSC			
***Chlamydia abortus***	P		P; FSC	B (ELISA)	P
***Leptospira* sp.**	F			B (MAT, ELISA)	F (kidney)
*Mycoplasma* sp. *	F	See PCR ^1^		B ( *M. bovis* ELISA)	FSC (culture / DGGE / PCR)
Tritrichomonas foetus*		VF, SW ^2^			
*Ureaplasma* sp. *	F	See PCR ^1^			FSC (culture / DGGE / PCR)
**Other bacteria (e.g.** ***Salmonella* sp., *Listeria* sp.,** ***Yersinia* sp., *Trueperella* sp.,** ***Bacillus* sp.)**	P, F	FSC			
***Neospora caninum***	F			B (ELISA)	F (brain)
***Toxoplasma gondii***	P, F			B (LAT)	P (cotyledon)
Bluetongue virus *	F			B (ELISA)	B, F (spleen, brain)
Bovine herpesvirus (BHV-1) *	F			B (ELISA)	F (liver, lung)
**Pestivirus (BD / BVD)**	P, F			B (Antibody ELISA; antigen ELISA for BVD)	F (spleen or thymus)
Schmallenberg virus *	F			B (ELISA)	B; F (brain, spinal cord); FF
**Mycoses (particularly ** ***Aspergillus* sp.)**	P, F	FSC (P)			

B, maternal blood; FF, fetal fluids; F, fetus; FSC, fetal stomach contents; P, placenta; SW, sheath washings; VF, vaginal fluids. MZN, Modified Ziehl-Neelsen stain of smear; ELISA, enzyme-linked immunosorbent assay; LAT, Latex Agglutination Test; MAT, microscopic agglutination test; RBT, Rose Bengal test. DGGE, denaturing gradient gel electrophoresis. * Pathogens not included in the standard abortion protocol. ^1^ For *Mycoplasma* sp. (including *Ureaplasma* sp.), a combined test comprising culture, DGGE, and PCR. ^2^ Tested by culture and microscopy Great Britain is officially brucellosis free ( *Brucella abortus*, *B. melitensis*, *B. ovis*). Ruminant abortion (including premature birth) submissions are screened for Brucella sp. as part of continued surveillance.

**Table 2c.** Abortion diagnosis in Germany ^1^ (reporting partner: FLI).

Pathogen / Test	Histopathology ^1^	Bacteriology (culture)	Serology	PCR or RT-PCR
***Coxiella burnetii***	F, P		S, Pm, M (ELISA)	F, FSC, LF, M, P, VS
***Brucella* spp.**	F, P		S (RBT, CFT, ELISA)	F, P, VS
***Campylobacter* spp. (small ruminants)**		F, LF, P, VS		
***Campylobacter fetus* subsp. *venerealis* (cattle)**		F, LF, P, PS, VS		
***Chlamydia abortus***	F, P		S (ELISA)	F, LF, VS, P
***Leptospira* sp.**	P		S (MAT)	F, VS
*Mycoplasma* sp . *		M ( *M. bovis*)	S, M (ELISA, *M. bovis*)	
*Tritrichomonas foetus* *		VS, PS		VS, PS
**Other bacteria (e.g. *Salmonella* sp *.*, *Listeria*** ** sp., *Yersinia* sp.,, *Trueperella* sp *., Bacillus* sp.)**		F, FO, P, VS		
***Neospora caninum***	F, P		S (ELISA)	F, LF
*Toxoplasma gondii* *				F (brain), P
Bluetongue virus *			S (ELISA)	B, S, F
**BHV-1 (cattle)**	F, P		S (ELISA)	LF, VS
**Pestivirus (BD / BVD)**	F, P		S (ELISA Ab+Ag)	B, M, NS (BVD), EP (BVD)
**Schmallenberg virus**	F, P		S (ELISA)	B, F (brain), LF, VS
Mycoses * (particularly *Aspergillus* sp.)		P, F		

B: maternal blood; EP: ear punch sample; F: fetus (organs); FSC *,* fetal stomach contents; LF: lochial fluid; M: milk; NS: nose swab; P: placenta; Pm: plasma; PS: preputial smegma; S: maternal serum; VS: vaginal swabs *.* CFT, complement fixation test; ELISA, enzyme-linked immunosorbent assay; MAT, microscopic agglutination test; RBT, Rose Bengal test. * Pathogens not included in the standard abortion protocol. ^1^ Histopathology is performed on request.

**Table 2d.** Abortion diagnosis in the Basque Country, Spain (reporting partner: NEIKER).

Pathogen / Test	Histopathology ^1^	Bacteriology		Serology	PCR or RT-PCR
		**Culture ^2^**	**MZN ^3^**		
***Coxiella burnetii***	F; P		P (F; VS)	S (ELISA)	P; F; VS
*Anaplasma phagocytophylum* *					B
***Brucella* sp.**	F; P	P; F; FSC; VS	P (F; VS)	S (RBT; CFT)	
*Campylobacter* sp. (small ruminants) *	F; P	P; F; FSC; VS			
*Campylobacter fetus* subsp. v *enerealis* *	F; P	P; F; FSC; VS; PS			VS; PS
***Chlamydia abortus***	F; P		P (F; VS)	S (ELISA)	P; VS; F
***Leptospira***	F; P			S; FF ^4^ (MAT)	F (lung; kidney); U; M
*Mycoplasma* *	F; P	P; VS			
*Tritrichomonas foetus* *	F; P	P; F; VS; PS			VS; PS
*Ureaplasma* *	F; P	P; F; VS			
**Other bacteria (e.g. *Salmonella* sp., ** ***Listeria* sp., *Yersinia* sp., *Trueperella* sp., ** ***Bacillus* sp.)**	F; P	P; F; FSC; VS			
***Neospora caninum***	F; P			S (ELISA)	P, F
***Toxoplasma gondii***	F; P			S (ELISA)	P, F
**Bovine herpesvirus (BHV-1)**	F; P			S (ELISA)	P, F
**Pestivirus (BD / BVD)**	F; P			S; FF ^4^ (ELISA)	P; F; B
Schmallenberg virus *	F; P			S (ELISA)	P; F; Me; B
Mycoses ( *Aspergillus*) *	F; P	P			

B: maternal blood, F: fetus, FF: fetal fluids, FSC *,* fetal stomach contents, M: milk, Me: meconium, P: placenta, PS: bull preputial smegma, S: maternal serum, U: urine, VS: vaginal swabs. MZN, Modified Ziehl-Neelsen stain of smear; CFT, Complement Fixation Test; ELISA, enzyme-linked immunosorbent assay; MAT, microscopic agglutination test; RBT, Rose Bengal test. * Pathogens not included in the standard abortion protocol. ^1^ Fetal organs taken for histopathology include the CNS (Pestivirus, Schmallenberg, *Toxoplasma* & *Neospora*), liver ( *Coxiella*, BHV1), kidney ( *Leptospira*); thyroid (Pestivirus), spleen (Pestivirus), lung ( *Coxiella*, BHV1, *Leptospira*); lip and eyelid (Pestivirus), skeletal and cardiac muscle (protozoa), and skin (Mycoses). ^2^ Fetus (F) for bacterial culture refers to liver. ^3^ For MZN the placenta is preferred; if not available, fetus (organ or stomach content) and/or vaginal swabs are analysed instead. ^4^ Serology of fetal fluids for immunocompetent fetuses.

**Table 2e.** Abortion diagnosis in The Netherlands (reporting partner: Royal GD).

Pathogen / Test	Histopathology ^1^	Immuno- histochemistry (IHC) ^1^	Bacteriology (culture)	Serology	PCR or RT-PCR
***Coxiella burnetii* ^2^**	F; P	F; P		S (ELISA)	F; P
***Brucella* sp. ^3^**				Mandatory sampling of maternal sera from aborted cattle (RBT)	
***Campylobacter* sp.**	F; P		FSC		
***Chlamydia abortus***	F; P	F; P		S (ELISA)	F; P
***Leptospira* sp.**	F; P	F; P		S (cattle)	
***Mycoplasma***				S ( *M. bovis* in cattle)	
**Other bacteria (e.g. *Salmonella*** ** sp., *Listeria* sp., *Yersinia* sp., ** ***Trueperella* sp *., Bacillus* sp.)**	F; P		FSC	*Salmonella* in cattle: S (ELISA)	
***Neospora caninum***	F; P			S (cattle)	
***Toxoplasma gondii***	F; P	F; P			F; P
Bluetongue (BTV) *	F			Fetal serum (ELISA)	F (if the fetus shows typical abnormalities)
**Bovine herpesvirus (BHV-1)**	F; P	F		S (IgE ELISA)	F
**Pestivirus (BD/BVD-small** ruminants *; BVD-cattle)	F			S (ELISA)	F (antigen ELISA)
*Schmallenberg* virus *	F			Fetal serum (ELISA)	F (if the fetus shows typical abnormalities)

F, fetus; P, placenta; S, maternal serum. ELISA, enzyme-linked immunosorbent assay; RBT, Rose Bengal test. * Pathogens not included in the standard abortion protocol unless the fetus shows typical abnormalities fitting BTV or SBV. ^1^ Fetal tissues taken for histopathology and IHC include: CNS; liver; lung cardiac muscle (protozoa). ^2^ In sheep and goats, coxiellosis is a notifiable disease with an increased number of abortions notification criterion. ^3^ *Brucella* is not included in the ruminant abortion pathology protocol since The Netherlands is free. Brucellosis has been investigated in cattle by compulsory testing of aborted cattle. For small ruminants, sheep and goats from 1,475 farms are tested annually by serology (RBT). More tests are not included in the table because samples are collected by the farmer: vaginal swabs of small ruminants for *Chlamydia abortus* PCR, deep throat swabs of aborted lambs/kids for PCR, and bacteriological examination for multiple causes of abortion.

**Table 2f.** Abortion diagnosis in Belgium (reporting partner: Sciensano-DGZ).

Pathogen / Test	Bacteriology		Serology	PCR	Other
	Culture	MZN			
***Coxiella burnetii***		P; F (SR ^1^)	S (ELISA – C ^2^, SR ^2^)	P; F (C ^2^, SR ^1^)	
*Anaplasma phagocytophilum* *				P; F (C ^2^)	
***Brucella* spp.**	P; F (C ^1^, SR ^1^ if MZN is positive)	P; F (SR ^1^)	S (SAW, MAT, ELISA – C ^1^, SR ^1^)		
***Campylobacter* spp.**	F (C ^2^, SR ^1^)				
***Chlamydia abortus, Chlamydia*** ** spp.**		P; F (SR ^1^)	S (ELISA *C. abortus* - SR ^1^)	*Chlamydia* spp.: P; F (C ^2^, SR ^1^ if MZN-positive)	
*Leptospira* sp. *			S ( *L. Hardjo* ELISA - C ^2^)	P; F (C ^2^)	
*Ureaplasma* sp. *				*U. diversum*: F (C ^2^)	
**Other bacteria (e.g.** ***Salmonella* sp *.*, *Listeria* sp.,** ***Yersinia* sp., *Trueperella* sp.,** ***Bacillus* sp.)**	F (C ^2^, SR ^1^)				
***Neospora caninum***			S (ELISA – C ^1^, SR ^2^); F-blotting paper (ELISA – C ^2^, SR ^2^)	F; P (C ^2^)	histopathology on F (heart and brain) – C ^2^
***Toxoplasma gondii***				F; P (SR ^1^)	
Bluetongue virus *			S (C ^2^, SR ^2^)	F – C ^2^, SR ^2^	
Bovine herpesvirus (BHV-4) *				F (C ^2^)	
**Pestivirus (BD / BVD)**					F (ear notch) antigen ELISA – C ^1^; S (if F tests positive) antigen ELISA – C ^1^
Schmallenberg virus *			S (C ^2^, SR ^2^)	F-C ^2^SR ^2^	

F, fetus; P, placenta; S, maternal serum. MZN, Modified Ziehl-Neelsen stain of smears. ELISA, enzyme-linked immunosorbent assay; MAT, microscopic agglutination test; SAW; Slow Agglutination of Wright. * Pathogens not included in the standard abortion protocol. ^1^ indicates that testing is part of the basic “Abortion Protocol” funded by FASFC; ^2^ additional tests for abortion diagnosis offered by DGZ and/or ARSIA and subject to a fee. The conditions applied to each animal species are as follows: cattle (C), sheep, and goats (SR). Other tests performed within the basic protocol funded by FASFC are necropsy of the fetus (cattle, sheep and goats) and general mycological examination of the fetus in sheep and goats. Other additional tests offered by DGZ and/or ARSIA and subject to a fee include histology, minerals, and vitamins (Se, I, Vit A, Vit E) on maternal sera, fetus (when suspected of Se or I deficiency), immunoglobulins, IgM, and serum amyloid A (SAA) in fetal thoracic fluid. If the fetus or afterbirth cannot be recovered, a dry vaginal swab is collected from the dam.

Methods for the diagnosis of *C. burnetii* infection are presented in Table 3. *C. burnetii* PCR is conducted by all (6/6) partners in cases of abortion in ruminants based on several matrices, mainly the placenta, fetal tissues, and vaginal fluids from aborted females. Histopathology of the fetus and placenta is performed by four out of six (4/6) partners, although only Royal GD combines this with immunohistochemistry (IHC) in cases of placentitis as a diagnostic tool in (small) ruminants. Bacteriology for *C. burnetii* is mainly based on microscopic examination of smears (placenta, fetal stomach content, and/or vaginal discharge) stained using modified Ziehl-Nielsen, and it is applied by four out of six (4/6) partners. The presence of antibodies against *C. burnetii* in maternal serum is available by serology in six out of six (6/6) partners, although different commercial ELISA kits were used.

**Table 3.** *Coxiella burnetii* investigation in abortion submissions by the different partners.

*Reporting* * Partner*	Histopathology	Bacteriology ^1^	Serology	Real-time PCR
**ANSES**			S (ELISA)	VS; P; F; FSC ^2^
**APHA**	P	P; FSC (MZN)	S ^3^ (ELISA)	P; FF ^4^
**FLI**		P; F; FSC; VS; M (culture)	S; Pm; M (ELISA)	P; F; FSC; VS; M
**NEIKER**	P; F ^5^	P; F; VS (MZN) ^6^	S (ELISA)	P; F; VS
**Royal GD**	P; F (including IHC ^7^)		S (ELISA)	VS ^8^; F; P (cattle)
**Sciensano**	F	P; F (MZN)	S (ELISA)	F ^9^; P; VS; B

B: maternal blood; BTM: bulk tank milk; F: fetus; FF: fetal fluid; FSC *,* fetal stomach contents; M: milk; P: placenta; Pm: plasma; S: maternal serum; VS: vaginal swab. IHC, immunohistochemistry; MZN, Modified Ziehl-Neelsen stain of smear; ELISA, enzyme-linked immunosorbent assay. ^1^ Bacteriology refers to MZN smears or culture isolation as stated. ^2^ For PCR, vaginal swabs are the preferred samples (essentially because they are optimal for preventing zoonotic transmission to the operators during shipment, storage, and analysis); when the test is performed on the placenta (collected in the uterus), a pool of three cotyledons is used; the test on the aborted fetus targets the spleen, liver, or stomach contents. Semi-quantitative PCRs (relative to a “clinical threshold”) or quantitative PCRs are performed. ^3^ Serology as ancillary test. ^4^ PCRs are performed when *C. burnetii* is detected or suspected (on MZN smears) and for all statutorily reported positive farms. ^5^ Fetal organs analysed are primarily liver and lung. ^6^ If placenta is not available, fetal (organ or stomach content) and/or vaginal swabs are analysed instead. ^7^ Immunohistochemistry is performed in case of placentitis. ^8^ Official protocol Dutch Food and Consumer Safety Authority in case of an increased number of abortions on small ruminant farms. ^9^ In the fetus, the abomasum, abomasum content, spleen, and liver are the preferred target, but all organs can be used. PCR is compulsory for small ruminants (part of the “Abortion protocol” funded by FASFC).

### 3.2 Interpretation of results of abortion submissions in relation to *Coxiella burnetii*

**Figure 1.**
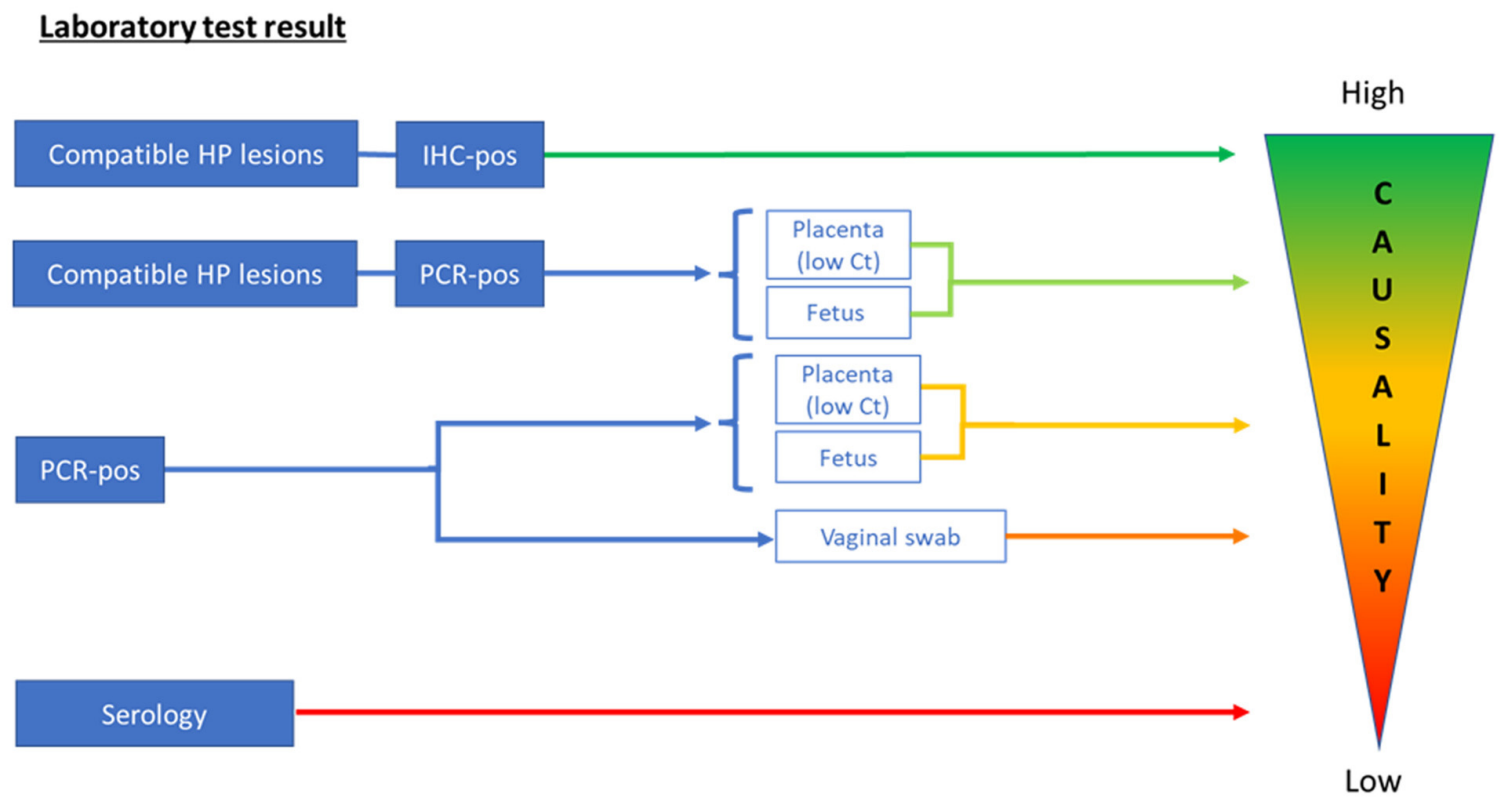
Interpretation of laboratory test results for *Coxiella burnetii* in relation to causality of abortion. Scheme of proposed interpretation of laboratory test results in relation to their value for the diagnosis of *Coxiella burnetii* as the cause of the abortion (causality). For immunohistochemistry (IHC) and histopathology (HP), both the fetus and placenta can be used, and their diagnostic values are comparable. Ct, Cycle threshold; Pos, positive.

The interpretation of *C. burnetii* results by different methods and in different sample matrices in relation to abortion in ruminants is presented in Table 4 and Figure 1. Samples that are mainly investigated by the consortium partners are placenta (including cotyledons), fetal samples (including fetal fluid), vaginal swabs, and maternal blood samples. The interpretation of results can be challenging and difficult to harmonize. Histopathology combined with IHC on placental membranes and cotyledons is considered the most reliable diagnostic technique for abortion diagnostics, but it is not as sensitive as real-time PCR.

**Table 4.** Interpretation of *Coxiella burnetii* results by different test methods and in different matrices/samples in relation to abortion in ruminants.

Technique	Sample	Prove of causality ^1^ (-, +/-, +, ++, +++)	Interpretation and conclusion
**MZN Smears**	Placenta/fetus	+	Unspecific result on its own; needs confirmation of presence of *Cb* by PCR.
**Histopathology (HP)** Typical pathological lesions and IHC positive	Placenta (fetus) ^2^	+++	Lesions compatible with *Cb* are not pathognomonic; needs positive IHC or IHC positivity to prove *Cb* causality of the abortion.
**Real-time PCR** In general, *Cb* PCR on its own does not prove a relationship between the detection of the pathogen and the abortion, although both the type of sample investigated and the level of positivity influence the likelihood of causality. The higher the level of positivity, the more likely causality. Causality can be proven by HP in combination with immunohistochemistry.			
	Fetus	++	Can be used to confirm the detection of *Cb*, in association with compatible histopathological findings. In the absence of compatible lesions, PCR positivity makes the relationship between pathogen and abortion more likely if other abortifacients have been ruled out.
	Placenta	++	Same interpretation as fetus but only if *Cb* PCR positivity level is high; low PCR positivity levels can be the result of sample contamination.
	Vaginal discharge	+	Indication for a putative relationship between abortion and *Cb* that needs further confirmation. Shedding of *Cb* can also occur after normal parturition.
	Bulk tank milk or individual milk sample (independently of Ct-value)	-	Detects shedding of *Cb* in a herd/flock or at the individual level. Does not prove a relationship between abortion and pathogen.
	Environmental sample dust/air/…) (independently of Ct-value)	-	Detects environmental contamination with *Cb* which may result in human exposure. Does not prove a relationship between abortion and pathogen. Tracing the most likely source of shedding may be advised depending on the context of the result.
**Serology ^3^**	Maternal serum	-	Seropositivity shows previous contact with *Cb*, but no causality with abortion. The relationship between shedding/abortion and serology is poor.
	Milk	-	

Abortion: Abortion, stillbirth, premature birth or weak offspring MZN: modified Ziehl-Neelsen stained smears (unspecific method) *Cb*: *Coxiella burnetii* HP: Histopathology IHC: immunohistochemistry ( *Cb* specific antibodies are currently unavailable for every routine laboratory). Ct: Cycle threshold ^1^ Prove of causality based on expert opinion ^2^ Placenta is more informative than the fetus ^3^ Positive serology can be a result of previous infection, vaccination or even cross-reaction

The most common PCR target used was the multicopy IS1111 element (used by 6/6 partners), as targeting a multicopy gene is anticipated to increase PCR sensitivity over a single-copy PCR target. PCR of the placenta, abomasal content of the fetus, and vaginal swabs was considered the most sensitive approach, particularly when IS1111 PCR was used. However, only when PCR is positive for placenta with relevant histopathological findings and/or fetal tissues, fetal fluid, or stomach contents from aborted fetuses test positive by PCR can *C. burnetii* be considered a potential cause of abortion. In other circumstances, causality can be suspected but not confirmed following a PCR-positive test. In these instances, other abortifacient agents need to be ruled out, and herd background and other laboratory results should be considered before reaching a diagnosis.

Serological results from maternal blood samples are even more difficult to relate to the cause of abortion, as not all infected animals seroconvert. In addition, positive serology (in non-vaccinated dams) provides information on previous exposure to *C. burnetii*, but not on the time of exposure; thus, causality cannot be established. Nevertheless, in some countries, serological sampling recommendations are used to investigate animals within a certain time period after they show reproductive disorders. Furthermore, it is currently not possible to differentiate between animals exposed to *C. burnetii* and those vaccinated against coxiellosis. In cases where *C. burnetii* is suspected to be the cause of abortion, additional sampling and/or testing should be conducted.

## Discussion

Diagnosis of the cause of abortion in ruminants is complex, and identification of the specific etiology is not always achieved ( Clune *et al.*, 2021; Hecker *et al.*, 2023). Success depends on the samples available for laboratory analyses, the techniques used, and the additional clinical and epidemiological information available (e.g., serial abortions, history of affected animals, stressful conditions, possible co-infections, and other investigations that point towards an abortive infection). Here, a comparison of ruminant abortion protocols submitted by all participants showed some variation in the way abortion diagnostics were performed. This enabled the discussion of the minimum and best practice requirements for the detection of abortifacient pathogens, including *C. burnetii*.

To establish the best standardized protocol for ruminant abortion diagnosis, it is essential to consider that these protocols may need to evolve as scientific knowledge and available tools advance. In addition, cost-benefit considerations play a crucial role. Each additional test will improve the likelihood of detecting an agent but will also increase costs. The pathogens to be investigated should be adjusted according to the current epidemiological situation of animal diseases and the evidence available in each region. Diagnosis requires detection of the pathogen, usually by isolation and/or PCR, with histopathology aiding diagnosis by identifying compatible lesions, which, in some cases, may be pathognomonic (e.g., neosporosis). Currently, the protocols for abortion investigation in ruminants contain a tailored combination of histopathology, bacteriology, serology, and/or real-time PCR for abortifacient agents known to be present in a certain region. If routine laboratory techniques fail to reach a diagnosis when an infectious cause is probable, further efforts should concentrate on detecting less likely or new pathogens. An advantage of post-mortem examination is that it allows the detection of new pathogens and novel histopathological changes ( Borel *et al.*, 2014; Hoffmann *et al.*, 2012; Van den Brom *et al.*, 2012; Van den Brom *et al.*, 2021). In the future, molecular techniques capable of identifying multiple agents simultaneously would improve the broadness of abortion diagnostics in ruminants, although interpretation of the findings would need to be done with caution ( Periyasamy *et al.*, 2025).

For the diagnosis of *C. burnetii* abortion, the most suitable samples are the placenta, fetus, and vaginal swabs from aborted animals, of which the latter have biosecurity advantages regarding transport and laboratory handling. The highest bacterial loads are found in placental membranes, with up to 10 ^9 ^bacteria per gram ( Babudieri, 1959; Fournier *et al.*, 1998). When placental samples are submitted, both intercotyledonary tissue and cotyledons should be included ( Roest *et al.*, 2012). *C. burnetii* replicates intensively within trophoblast cells of the placenta; these cells are present in the villi of cotyledons and in smaller numbers in the intercotyledonary tissue. It is important that the veterinarian collects appropriate samples from a fresh placenta and submits them according to laboratory requirements. The fetal samples of choice were the spleen, lung, liver, and abomasal contents (fetal stomach contents). Of these, the stomach content is the most practical sample to be collected. Furthermore, *C. burnetii* infection can progress in stages within fetal organs and may colonize the spleen before other organs. This suggests that multiple organs may need to be tested for fetal diagnosis, as non-detection of *C. burnetii* in one organ does not rule out its presence in other organs at any given time.

Histopathology of the fetus and placenta can identify lesions compatible with *C. burnetii* infection ( Agerholm, 2013), but the placenta and many fetuses will have some degree of autolysis, which will compromise histopathological examination. As histopathological changes alone are not pathognomonic for *C. burnetii*, suspected lesions need to be further corroborated by additional staining of histological samples (e.g., IHC). Thus, histopathology combined with IHC of the placenta and fetus is a useful reference for establishing the role of *C. burnetii* as the cause of abortion in ruminants, although *C. burnetii* specific antibodies for IHC are not easily available in all routine laboratories.

PCR is a sensitive and specific method for detecting *C. burnetii*. PCR can also be used to confirm the presence of *C. burnetii* in samples with positive MZN smears (an unspecific result on its own), and when compatible histopathological lesions are observed in placenta/fetal samples, and IHC is not performed. Real-time PCR analysis of the placenta, fetal tissues, and vaginal swabs (from aborted animals within eight days post-abortion) was used by all partners to test for *C. burnetii*. However, causality of abortion cannot be proven based solely on the detection of *C. burnetii* DNA by PCR, although this is more likely when the estimated bacterial loads are high. The observation of low cycle threshold (Ct) values from real-time PCR is indicative of high bacterial loads, but caution must be taken since an “Inter-laboratory proficiency testing programme report” revealed significant differences in Ct values obtained by different laboratories ( Rousset, 2021). The inclusion of a standard of known bacterial gene copy numbers in each PCR reaction could help control intra- and inter-laboratory variations in qPCR.

PCR tests targeting IS1111 are more sensitive than PCR tests designed to detect single-copy genes ( EFSA, 2010; Jones *et al.*, 2011). Because of this high sensitivity, PCR results with low bacterial load should be interpreted with care, as they can be the result of environmental contamination rather than shedding by the animal. In addition, because the IS1111 element is present in variable numbers in different *C. burnetii* strains, from seven to 110 copies according to Klee *et al.* (2006), the sensitivity of this method is biased towards samples that contain *Coxiella* isolates with higher IS1111 copy numbers. A French expert group has proposed a quantitative PCR threshold above which *C. burnetii* can be considered the likely cause of abortion, a guideline adopted at the European level ( EFSA, 2010) that French partners are currently applying. Under this proposal, a causative relationship between abortion in ruminants and *C. burnetii* is considered highly probable when at least 10 ^4^ bacteria per gram of placenta or vaginal swab is detected. However, this threshold may be more appropriate for vaginal swabs than for placental tissue, where bacterial loads are often much higher because of the intense replication of *C. burnetii* in trophoblasts. Therefore, a higher threshold for placental samples might be considered to rule out contamination. The *C. burnetii* strain *Nine Mile* RSA493, which displays 20 copies of the IS1111 element, is considered as a reference for copy number estimation ( EFSA, 2010). However, the use of a quantitative PCR threshold based on IS1111 amplification is debatable because the copy numbers of the target vary among *C. burnetii* strains. Accurate quantification of the bacterial load would require the use of a single-copy gene, such as *com1* or *icd*, as the target; however, its use in routine analyses is hampered by a loss in sensitivity.

Serology detects antibodies against a specific agent following previous exposure of the sampled animal, but cannot discriminate between vaccinated and naturally exposed individuals. However, the presence of antibodies in individual maternal serum does not prove a relationship between abortion and a particular agent. In the case of *C. burnetii*, the relationship between positive serology and shedding of *C. burnetii* is poor at the individual level ( Hogerwerf *et al.*, 2014; Rousset *et al.*, 2009). Several factors have contributed to this complexity. First, some animals may not generate an antibody response that is sufficiently strong to be detected by serological tests, falling below the detection threshold. In addition, some animals may exhibit delayed seroconversion, with antibody levels rising slowly and crossing the detection threshold one to two weeks after abortion. This delayed seroconversion can lead to false-negative results if samples are collected too early ( Arricau-Bouvery *et al.*, 2005; de Cremoux *et al.*, 2012; Rousset *et al.*, 2009). Consequently, the usefulness of serology for the diagnosis of abortions in individual animals is limited. However, at the herd level, seroprevalence levels of approximately 50% of the sampled animals may be indicative of active *C. burnetii* infection, requiring further testing for confirmation ( EFSA, 2010). Thus, serology performed at the herd level provides valuable complementary information to the results of direct diagnostic methods (IHC and real-time PCR) performed on aborted individuals, but is insufficient to diagnose *C. burnetii* as the cause of abortion in individual cases.

In several countries, vaccination against Q fever with a non-DIVA (Differentiating Infected from Vaccinated Animals) compatible vaccine is applied, which should be considered when serology results are interpreted. In addition, the sensitivity and specificity of ready-to-use commercial kits should be considered because recent studies have revealed that significant variations exist between available ELISAs depending on the ruminant species ( Lurier *et al.*, 2021).

Although coxiellosis frequently results in clinical signs (the ASPW complex), particularly in small ruminants, shedding of *C. burnetii* also occurs in the absence of clinical signs after normal parturition ( Álvarez-Alonso *et al.*, 2020). Therefore, notification and investigation of ruminant abortions will not detect all possible sources of *C. burnetii*. Additional surveillance systems are required to detect shedding, subsequent environmental contamination, and human exposure. In the case of asymptomatic infection, detection of shedding in dairy flocks/herds is possible by the collection and testing of bulk tank milk (BTM) ( Boarbi *et al.*, 2014; Jansen *et al.*, 2022; Van den Brom *et al.*, 2015), a method routinely used in the laboratories of two out of six (2/6) partners. Environmental samples (dust and aerosols) can also be tested for *C. burnetii* by PCR, but positive results only provide information on the presence of the bacterium in the tested sample/material, and not on the source of environmental contamination with *C. burnetii* ( Carrié *et al.*, 2019; De Bruin *et al.*, 2013; Hurtado *et al.*, 2017). In addition, PCR detection in the environment provides no information on viability or infectivity, which requires *in vitro* and/or *in vivo* infection models ( Mori *et al.*, 2013). However, it may prove to be a useful indicator, as it suggests that subsequent human and animal exposures are likely. Depending on the epidemiological context, such environmental indicators may highlight the importance of source tracing and additional measures to prevent further shedding ( Hurtado *et al.*, 2023). Additionally, human syndromic surveillance (where clusters of suspected Q fever cases are identified) in combination with geographic information system analyses can also help detect possible sources of *C. burnetii* shedding animals ( van den Wijngaard *et al.*, 2011).

## Conclusion

In conclusion, it appears that abortion diagnosis protocols for ruminants are tailored by each of the partners, based on the infectious agents present in each region. Histopathology, including IHC, on fetal and placental membranes supplemented with bacteriology, serology, and real-time PCR, is currently used for the detection of infectious causes of abortion in ruminants. This is the broadest diagnostic approach to relate a detected agent to the pathological changes in the fetus and/or placenta. Although harmonization of protocols in terms of sample and test selection may seem desirable, it is not without limitations that include both cost and organizational considerations. For the identification of *C. burnetii* as the causative agent of abortion, based on the consortium expert opinion, we provide some guidelines for the interpretation of laboratory test results in relation to their diagnostic value. PCR is the preferred diagnostic tool for the detection of *C. burnetii* shedding because it is very sensitive. However, because of this high sensitivity, real-time PCR results with low bacterial load should be interpreted with caution because they can be the result of environmental contamination rather than shedding by the animal.

Despite advances in diagnostic techniques, establishing a standardized protocol that balances sensitivity, specificity, practicality, and cost remains challenging. In the case of *C. burnetii*, future efforts should focus on harmonizing diagnostic protocols across regions and improving our understanding of its role in ruminant abortion to improve control and prevention strategies.

## Ethics statement

Ethical approval and consent were not required.

## Data Availability

No additional data are associated with this article.
