# Supplementary material for: Detection of abortifacient agents in domestic ruminants, with a specific focus on Coxiella burnetii
Source: Open Res Eur. 2025 Jun 30;5:94. Originally published 2025 Mar 31. [Version 2] doi: 10.12688/openreseurope.19270.2 (PMC12340485; doi:10.12688/openreseurope.19270.2)
Supplement: Peer review history

# Reviewer report: Reviewer response for version 2

**DOI:** 10.21956/openreseurope.22435.r56274

**Published:** 2025-08-11

**Authors:** George Peter Semango

**Affiliations:** 1 Nelson Mandela African Institution of Science and Technology, Arusha, Tanzania

Thank you for the revised version of the manuscript. This version looks much better in terms of the references and flow. Well done!

Is the study design appropriate and does the work have academic merit?

Yes

Is the work clearly and accurately presented and does it cite the current literature?

No

If applicable, is the statistical analysis and its interpretation appropriate?

Not applicable

Are all the source data underlying the results available to ensure full reproducibility?

Yes

Are the conclusions drawn adequately supported by the results?

Yes

Are sufficient details of methods and analysis provided to allow replication by others?

Yes

Reviewer Expertise:

One Health, Biomedical sciences

I confirm that I have read this submission and believe that I have an appropriate level of expertise to confirm that it is of an acceptable scientific standard.

# Reviewer report: Reviewer response for version 1

**DOI:** 10.21956/openreseurope.20859.r54454

**Published:** 2025-05-27

**Authors:** Richard Nyamota

**Affiliations:** 1 International Livestock Research Institute, Nairobi, Nairobi County, Kenya

**Introduction: **The title broadly suggests a focus on the diagnosis of abortifacient pathogens; although the authors imply a focus on *Coxeilla burnetii,* the manuscript quickly narrows down to *Coxiella burnetii* without acknowledging the broader spectrum of these agents. It would strengthen the introduction to briefly outline the range of abortifacient pathogens before focusing on *C. burnetii*. In the introduction, four work packages are described but it is not clear to me whether this review was within one of the described work packages. Could be helpful to clarify this .

**Methods: **It would be helpful to include protocols from the different laboratories involved—perhaps as supplementary material—for transparency and to aid reproducibility."

**Discussion: **The discussion is well written and effectively highlights key diagnostic considerations for *C. burnetii*, including appropriate sample selection and the sensitivity of various markers, along with their limitations. In the conclusion, it would have been valuable for the authors to propose a hypothesis or framework for diagnostic method standardization. This could serve as a strong take-home message and provide a foundation for future efforts toward harmonizing protocols.

Is the study design appropriate and does the work have academic merit?

Yes

Is the work clearly and accurately presented and does it cite the current literature?

Partly

If applicable, is the statistical analysis and its interpretation appropriate?

Not applicable

Are all the source data underlying the results available to ensure full reproducibility?

No

Are the conclusions drawn adequately supported by the results?

Yes

Are sufficient details of methods and analysis provided to allow replication by others?

Partly

Reviewer Expertise:

One health, disease surveillance, pathogen discovery, genomic characterisation and molecular epidemiology

I confirm that I have read this submission and believe that I have an appropriate level of expertise to confirm that it is of an acceptable scientific standard.

# Reviewer report: Reviewer response for version 1

**DOI:** 10.21956/openreseurope.20859.r53093

**Published:** 2025-05-10

**Authors:** George Peter Semango

**Affiliations:** 1 Nelson Mandela African Institution of Science and Technology, Arusha, Tanzania

A wonderful paper outlining the detection of abortifacients in domestic ruminants with a focus on C. burnetii. Very relevant and interesting

 Introduction

 1. Quite old references. Any references less than 10 years?

 2. C. burnetii should always be in Italics.

 3. Perhaps an introductory paragraph of the partner Institutions/ Centres that are in the methods section? Perhaps in the consortium partners paragraph.

 4. Workpackage paragraph should perhaps include respective partner centres for each of the workpackages.

 Methods

 1. A non-European reader would not know the location of the reference labs (ANSES, APHA etc). Perhaps an introductory paragraph in the introduction.

 Discussion

 The discussion leans towards modern technology. However, quite old references are used giving a distorted picture that perhaps the paper is leaning towards outdated ideas.

Is the study design appropriate and does the work have academic merit?

Yes

Is the work clearly and accurately presented and does it cite the current literature?

No

If applicable, is the statistical analysis and its interpretation appropriate?

Not applicable

Are all the source data underlying the results available to ensure full reproducibility?

Yes

Are the conclusions drawn adequately supported by the results?

Yes

Are sufficient details of methods and analysis provided to allow replication by others?

Yes

Reviewer Expertise:

One Health, Biomedical sciences

I confirm that I have read this submission and believe that I have an appropriate level of expertise to confirm that it is of an acceptable scientific standard, however I have significant reservations, as outlined above.
